# Supplementary material for: Colocalization between bone loading and metabolic activity in the human tibia in response to acute exercise
Source: J Bone Miner Res. Author manuscript; Available in PMC 2026 Nov 16. (PMC13772117; doi:10.1093/jbmr/zjag163)
Supplement: Supplementary file 1 [file NIHMS2220972-supplement-Supplementary_Information.pdf]

Supplementary Information

Colocalization between bone loading and metabolic activity in the human tibia in response to acute exercise

Olivia L Bruce, PhD[a], Ananya Goyal, MSc[a,b], Yael Vainberg, BSc[a], Anthony A Gatti, PhD[a], Feliks Kogan, PhD[a]

a Department of Radiology, Stanford School of Medicine, Stanford, California, USA

b Department of Bioengineering, Stanford University, Stanford, California, USA

This is the Authors' Accepted Manuscript of an article published in the Journal of Bone and Mineral Research. The Version of Record is available at JBMR's website.

Authors Accepted Manuscript

Orthoload Data

List of files from the Orthoload database (https://orthoload.com/database/) used to compute mean knee contact forces and moments at the time of peak resultant knee contact force during stair descent.

k1l_110108_2_37p

k2l_301107_1_144p

k3r_250309_1_105p

k4r_201108_2_3p

k5r_060809_1_56p

Bergmann G. and Damm P. (ed.), Julius Wolff Institute, Berlin Institute of Health at Charité Universitätsmedizin Berlin (2008) "OrthoLoad".

Supplemental Figures

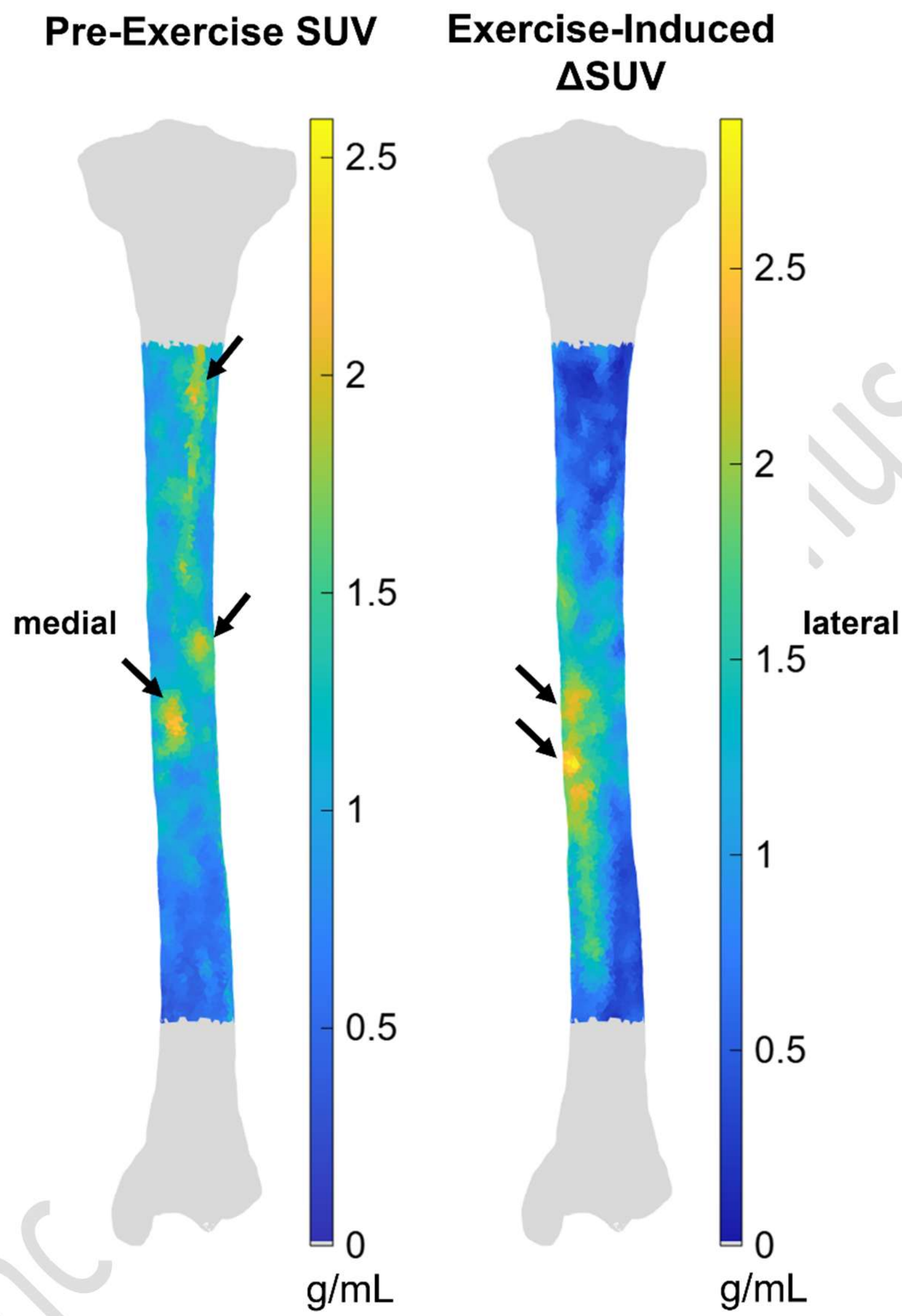

Author Manuscript

Figure S1: Posterior view of the tibia of a representative participant with colormaps of pre-exercise SUV (left) and exercise-induced change in SUV (right) mapped to the diaphysis. In both images, small regions of elevated SUV (or change in SUV) are observed on the posterior surface, indicated by arrows. Small regions of increased SUV were observed on the posterior surface were observed in approximately 50% of tibiae.

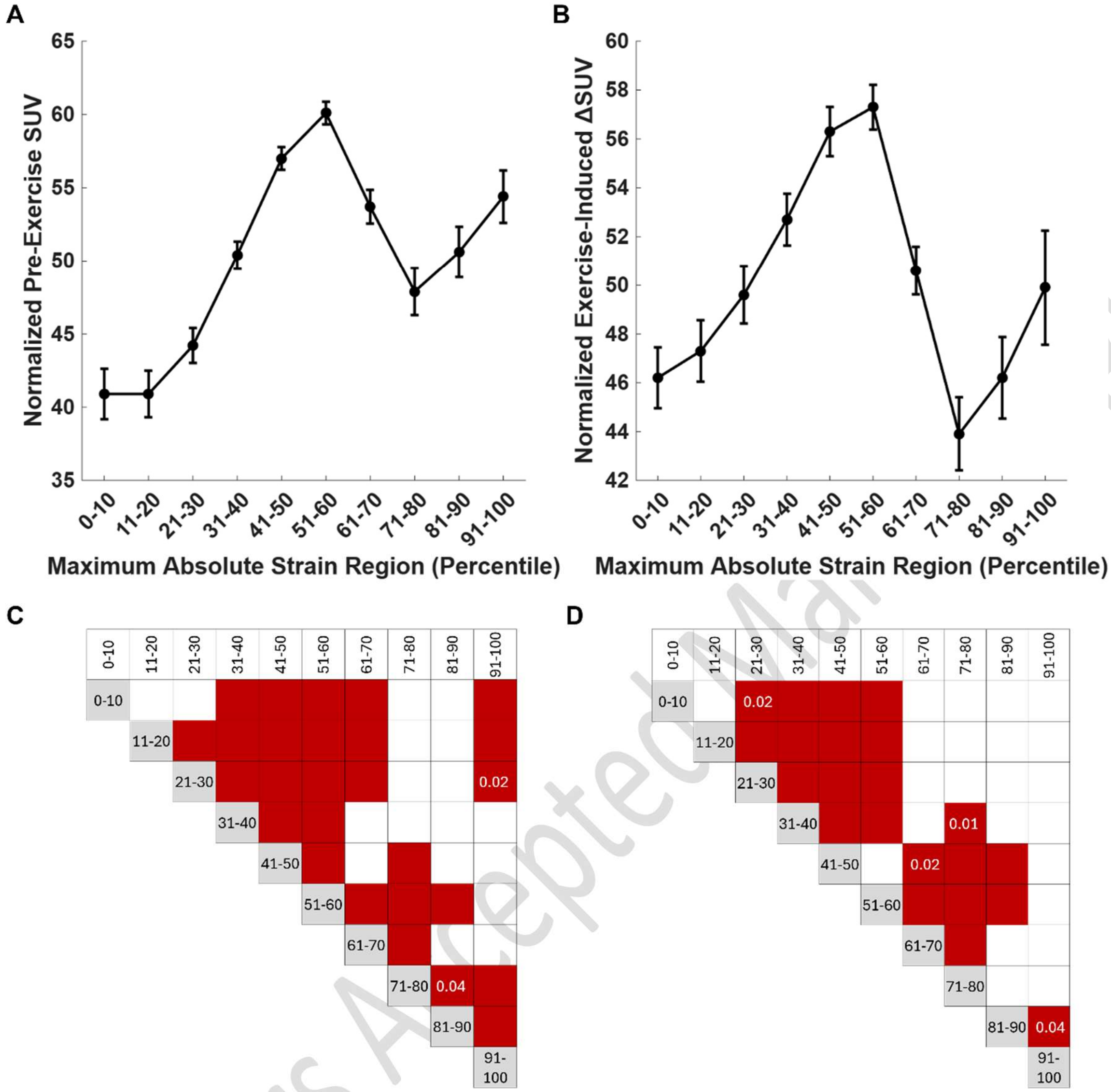

Figure S2: Estimated marginal mean and standard error of (normalized) SUV across maximum absolute strain regions. Top: Mean pre-exercise SUV and exercise-induced change in SUV did not show a directional relationship with compressive (minimum principal) strain. Bottom (C and D): Corresponding charts of pairwise comparisons of SUV between strain regions (x- and y- axes). Significant comparisons are shaded red. p-values were < 0.01 unless otherwise shown in each red square.

Authors Accepted Manuscript

Post-hoc power analysis

We performed post-hoc assessments to evaluate power. Cohen's f^2 was calculated using the adjusted r^2 for each overall model. Effect size was very large for tensile strain regression models (f^2 = 1.50-2.45) and compressive strain regression models (f^2 = 0.61 – 0.69).

Kish's design effect was then calculated to evaluate how clustering by participant (potential correlations between observations from regions within the same bone and left/right legs) might affect the effective sample size. Intra-class correlation coefficients (3,1) were calculated to measure the variance of measures within participants (20 samples per participant across regions and legs) relative to the variability of measures between participants. For all outcome measures, ICC was 0. Normalization of strain and SUV within each bone likely reduced variance between participants. As a result, Kish's design effect was equal to 1; the effective sample size was the same as the actual sample size.

Post-hoc calculations of achieved power for linear multiple regressions (fixed model, r^2 increase) were then calculated in G*Power (v3.1.9.7; Faul et al., 2009) with parameters: f^2 = 0.61, 0.69, 1.50, 2.45, alpha = 0.05, total sample size = 49, tested and total number of predictors = 11. Achieved power was 93 – 100%.

Authors Accepted Manuscript
